# The Yellow Fever Question

**Published:** 1879-01

**Authors:** 


### THE YELLOW FEVER QUESTION.

The Public Health Association, which recently has been in convention at Richmond, were engaged chiefly in the consideration of the nature, origin, mode of propagation, the contagiousness, and the proper treatment of yellow fever. A commission of competent medical men, appointed under the supervision and authority of the general government, proceeded in the hight of the epidemic to New Orleans to investigate its pathology and, if possible, lay a foundation for its rational treatment.

The epidemic has ceased, the work of the commission is done, and their report to the Association has been formally made. After a good deal of discussion, and the advancement, from different quarters, of a variety of opinions and hypotheses respecting the true character of the fever, the decision finally arrived at by the convention crystallized into the shape of the following conclusions:

*First.*—That the yellow fever of 1878 was a specific disease not indigenous to, nor originating spontaneously in the United States, and its appearance in this country during that year was due to specific causes.

*Second.*—That quarantine, established with such vigor and precision as to produce absolute non-intercourse, will prevent the importation of specific causes of yellow fever.

*Third.*—That it is the duty of the general government to aid in the establishment of practical and proper quarantine by all the means in its power.

*Fourth.*—That it is the duty of the general government to appoint a commission of experts to make a thorough investigation into the causes of yellow fever and the best method of preventing its introduction into this country, and to make such an appropriation as will permit of the securing of the services of the best men and of the best means for carrying out such an investigation.

*Fifth.*—That it is the duty of the general government to invite foreign nations to co-operate with it in the establishment of uniform and effective international quarantine regulations.

*Sixth.*—That whatever may be the permanent value of quarantine, there is no doubt of the importance and value of internal sanitary measures in the prevention or modification of epidemic yellow fever; and that this association urges upon State and municipal authorities the great amount of responsibility which rests upon them on this account at times when no disease is prevailing or threatening.

